# Meningococcemia Presenting as a Myocardial Infarction

**DOI:** 10.1155/2015/953826

**Published:** 2015-11-11

**Authors:** Daniel Lachant, David Trawick

**Affiliations:** Division of Pulmonary and Critical Care Medicine, University of Rochester Medical Center, 601 Elmwood Avenue, Rochester, NY 14624, USA

## Abstract

*Neisseria meningitidis* is an encapsulated gram negative diplococcus that colonizes the nasopharynx and is transmitted by aerosol or secretions with the majority of cases occurring in infants and adolescents. Meningococcemia carries a high mortality which is in part due to myocarditis. Early recognition and prompt use of antibiotics improve morbidity and mortality. We report a 55-year-old male presenting to the emergency department with chest pain, shortness of breath, and electrocardiogram changes suggestive of ST elevation MI who developed cardiogenic shock and multisystem organ failure from* N. meningitidis*. We present this case to highlight the unique presentation of meningococcemia, the association with myocardial dysfunction, and the importance of early recognition and prompt use of antibiotics.

## 1. Introduction

*Neisseria meningitidis* is an encapsulated gram negative diplococcus that normally colonizes the nasopharynx of humans and is transmitted by aerosol or secretions [1]. The annual incidence of meningococcal infections is estimated at 1,500–3,000 cases per year in the United States, typically occurring in the late winter and early spring [1]. Although infants and adolescents account for the majority of cases, adults over thirty comprise up to 30% of infections [1, 2]. Risk factors for infection acquisition include terminal complement deficiency, immune deficiencies, crowded living spaces [1], eculizumab [3], and asplenia [4]. Mortality ranges from 9% to as high as 40% in meningococcemia [1]. Myocarditis has been associated with meningococcemia in both children and adults and is associated with increased mortality [5, 6]. We report a unique case of meningococcemia presenting as chest pain and ST elevations on ECG mimicking an acute anterior myocardial infarction without any preceding infectious symptoms in a 55-year-old male.

## 2. Case

**Figure 1:**
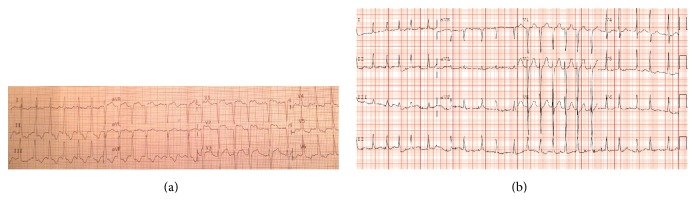
(a) Electrocardiogram from EMS showing ST elevation in V1–V3 with ST depressions in V4–V6, I, II, II, and AVF in the setting of chest pain. (b) Electrocardiogram 3 days later with resolution of ST changes.

A 55-year-old male with history of smoking and substance abuse presented to the emergency department (ED) with acute onset worsening chest pain and shortness of breath of 18-hour duration. He had no complaints of subjective fever, cough, sputum production, sweats, neck pain, or headache. On presentation, his core temperature by Foley catheter was 35.7°C, blood pressure 110/80 mmHg, respiratory rate 28 breaths per minute, heart rate 134 beats per minute, and hemoglobin saturation on 100% oxygen 95%. He was noted to be anxious and to have cool mottled skin; the remainder of his exam was benign. His initial ECGs performed by EMS and in the emergency department, respectively, demonstrated atrial fibrillation with a rapid rate and ST elevation in V1–V3 (Figure 1). The Myocardial Infarction Team was called as there was concern that the patient was experiencing an acute anterior ST elevation myocardial infarction. He was taken for emergent rescue percutaneous intervention before his initial blood work came back. The left heart catheterization revealed no obstructing lesion, no dissection, and an ejection fraction of 20%; his echo from one year previously demonstrated a normal ejection fraction. A concomitant right heart catheterization (RHC) revealed a right atrial pressure of 15 mmHg, a pulmonary artery occlusion pressure of 28 mmHg, systemic vascular resistance of 1172 dynes/sec/cm^5^, and a cardiac index of 2.68 liters per minute per meter squared. The pulmonary artery catheter was not left in and his hemodynamic parameters were not trended.

During the catheterization the initial labs drawn in the ED returned. The arterial blood gas was 7.21/34/161, serum lactate 6.6 millimoles/L, troponin <0.01 ng/mL (it was not trended but on day 2 it was 1.26 ng/mL), bicarbonate 14 millimoles/L, creatinine 2.17 mg/dL, INR 2.4, aPTT 52.5, fibrinogen 103 mg/deciliter, white blood cell count 7,900/*μ*L (with 14% bands), hemoglobin level 12.1 g/dL, and platelets count 17,000/microliter.

There was now concern for severe sepsis of unknown etiology, and vancomycin, piperacillin-tazobactam, and azithromycin were initiated 3 hours after admission. Five hours after the antibiotic administration, the patient developed refractory hypotension despite fluid resuscitation. Vasopressors were initiated including norepinephrine (peaking at 70 mcg/min within 12 hours of starting), vasopressin 0.04 units/min was started after norepinephrine reached 30 mcg/min (this is the dosing used at our institution, and it is not titrated), and hydrocortisone 50 mg was given every 6 hours. He subsequently developed respiratory failure shortly after vasopressors were started and was intubated. A CT chest/abdomen/pelvis showed possible multifocal pneumonia versus pulmonary edema. A gram stain of his sputum after antibiotics initiation revealed a few gram negative diplococci and gram positive cocci. Sixteen hours after presentation, his echocardiogram now showed global biventricular systolic dysfunction with an ejection fraction of 18% and cardiac index calculated to be 1.32. The patient developed persistent metabolic acidosis requiring high bicarbonate and calcium infusions. Following consultation with nephrology, the MICU team initiated continuous venovenous hemodialysis to better address the evolving acute renal failure, hypocalcemia, and metabolic acidosis.

Within 24 hours after admission one blood culture turned positive for gram negative diplococci eventually identified as* Neisseria meningitidis* Group A. High dose ceftriaxone was added and continued for 10 days. An LP was never performed due to his coagulopathy and hemodynamic instability. CT and MRI of his head did not show evidence of infection or bleed. Vasopressors were weaned off in 4 days and steroids were stopped 6 days after presentation, respectively.

By day 11, a repeat echocardiogram revealed that his ejection fraction had improved to 42% and a month later it went back to 55%. After his critical illness resolved he returned and remained in normal sinus rhythm. The areas of mottling on admission were demarcated at both hands and all of his toes developing gangrene ultimately requiring left transmetatarsal amputation, left transradial amputation, and partial right hand amputation. After working with physical therapy he is able to ambulate and requires assistance with activities of daily living. His renal function never recovered and he remains on dialysis.

## 3. Discussion

This is a unique case of a 55-year-old male with an intact immune system with meningococcemia without meningitis who presented after 24 hours of chest pain in the absence of an infectious prodrome and was noted to have an ECG strongly suggestive of an acute anteroseptal myocardial infarction. The chest pain and ECG changes were likely from myocarditis leading to congestive heart failure and cardiogenic shock as his coronary angiogram at the time was normal. In previous reports detailing meningococcemia with associated ST segment elevation the patients presented with an infectious prodrome which was absent in our patient [7–9]. However, similar to our patient, the previous cases also had elevated troponins, decreased left ventricular function on echocardiogram, developed shock requiring vasopressor and/or inotropic support, and angiograms which revealed normal coronary arteries without obstructing lesions [8, 9].

The causes of the ECG changes associated with meningococcemia are not known but are probably multifactorial, and some authors postulate transmural ischemia [7]. The incidence of myocardial ischemia is increased in acute meningococcemia in pediatric patients and correlates with myocardial dysfunction. Early recognition of myocardial injury allows for myocardial support and early replacement therapy with PC, AIII, factor VIII, or fibrinogen that might improve outcome in acute meningococcemia in children [6]. Myocarditis probably plays a role as autopsy series suggests associated rates of 27–57% in children and as high as 85% in adults [5]. Acute myocarditis, in general, may present with chest pain, ECG changes, and elevated serum markers, all of which were seen in our case [10] and may trigger coronary spasm mimicking myocardial infarction [11].

Septic shock, in general, is characterized by peripheral vasodilatation and hypotension. Sepsis-associated myocardial dysfunction has been known for years but is not always readily evident due to the concomitant elevated cardiac index [12]. However, the cardiovascular response to meningococcemia may be fundamentally different than what is seen with other forms of gram negative sepsis. Monsalve and colleagues retrospectively reviewed the hemodynamic data of patients admitted to their ICU with shock from* Neisseria meningitidis*; hemodynamic data of 19 of them were obtained within the first 6 hours [13]. These were compared to 20 patients admitted with shock from other gram negative organisms who underwent similar hemodynamic monitoring. Before volume expansion, the* Neisseria* group had a cardiac index of 2.3 ± 0.07 L/min^2^ and systemic vascular resistance of 1338 ± 205 dynes/sec/cm^5^ versus 3.5 ± 0.6 L/min^2^ and 826 ± 207 dynes/sec/cm^5^, respectively. The hemodynamic data from the* Neisseria* group in this study is very similar to what we found on right heart catheterization in our patient and further underscores why our case first appeared to be a cardiogenic rather than septic event. After the RHC the pulmonary artery catheter was removed and we were unable to trend further hemodynamic numbers as his shock progressed and improved.

The cardiac dysfunction occurs earlier compared to other gram negative bacteremia and typically precedes manifestations of shock [13]. In our patient, the RHC demonstrated a low cardiac index and high systemic vascular resistance during a time when the patient was normotensive despite a concomitant metabolic profile suggesting a metabolic acidosis with acute kidney injury and a complete blood count demonstrating a significant bandemia.

Previous studies have shown that TNF*α* and interleukin 1*β* contribute to myocardial dysfunction in sepsis [14]. However, in meningococcal sepsis, interleukin 6 appears to play more significant role than the one that has previously been ascribed to it [15]. Furthermore, there appears to be a significant relationship between interleukin 6 levels and disease severity in meningococcal disease and the levels often remain elevated for up to 48 hours after disease presentation which could explain the persistence noted in cardiac dysfunction following the onset of sepsis [16–19].

From our case and review of the literature, we believe the hypotension in meningococcal-induced shock is due, at least initially, to cardiogenic rather than distributive factors. The initial hemodynamic support measures may require more attention focused on inotropic support rather than vasopressors as the latter could theoretically contribute to vascular ischemia and subsequent gangrenous necrosis often seen in severe meningococcemia. In suspected cases of shock caused by meningococcemia, consideration should be given to using a pulmonary artery catheter to help elucidate cardiogenic versus distributive shock and help guide decisions regarding inotropic versus vasopressor therapy. In some cases, intra-aortic balloon pumps have been used successfully as an initial support measure [20]. Whether more aggressive use of inotropic support in our patient would have prevented or ameliorated his vascular complications is not known.

In conclusion we present this case of meningococcemia with a unique presentation of chest pain, ST elevation on ECG, and cardiogenic shock and without neurologic involvement or any preceding infectious symptoms. Prompt recognition and early use of antibiotics are the mainstay of treatment for this rapidly progressive infection given its high morbidity and mortality. Furthermore, as the initial hypotension in meningococcemia may be more cardiogenic than distributive, invasive hemodynamic monitoring with a pulmonary artery catheter may better guide hemodynamic management. In retrospect, the combination of mottled skin and low cardiac output with elevated right sided pressures could serve as a clue to possible meningococcal etiology if other lab data suggests infection. As the incidence of adults with meningococcemia presenting without neurologic involvement is extremely low, physicians are not typically thinking about this as a cause of cardiogenic shock especially in the presence of ST segment elevation.
